# Clinicopathologic characteristics and prognostic factors for primary spinal epidural lymphoma: report on 36 Chinese patients and review of the literature

**DOI:** 10.1186/s12885-017-3093-z

**Published:** 2017-02-14

**Authors:** Le Xiong, Ling-Min Liao, Jian-Wu Ding, Zhi-Lin Zhang, An-Wen Liu, Long Huang

**Affiliations:** 1 Department of Oncology, The Second Affiliated Hospital of Nanchang University, 1 Minde Road, Nanchang, Jiangxi China; 2 Department of Ultrasound, The Second Affiliated Hospital of Nanchang University, Nanchang, China; 3 Department of Otorhinolaryngology, The Second Affiliated Hospital of Nanchang University, Nanchang, China

**Keywords:** Primary spinal epidural lymphomas, Treatment, Prognosis

## Abstract

**Background:**

Due to the uncommon nature of primary spinal epidural lymphomas (PSELs), there has been little research looking at prognostic indicators for the tumor. To our knowledge, this is the largest study to evaluate possible clinical and pathologic prognostic factors in PSEL patients.

**Methods:**

We retrospectively reviewed 130 cases of PSEL, including 36 Chinese patients and 94 published case reports from 1985 to 2015. Patient treatment regimens included surgery (S; *n* = 119), surgery followed by chemotherapy (S + CT; *n* = 25), surgery followed by radiotherapy (S + RT; *n* = 26), and surgery followed by chemotherapy and radiotherapy (S + CT + RT; *n* = 50).

**Results:**

Review of the most recent case follow-up data (time varied) found 51 patients (47%) alive and tumor-free, 10 patients (9%) alive with tumor present, and 47 patients (44%) deceased. The 3-year overall survival (OS) and disease-free survival (DFS) rates were 81.1% and 46.3%, respectively. Favorable prognostic factors found by univariate analysis were female sex, B-cell lymphoma diagnosis, cervical spine location, and combined modality treatment. Furthermore, multivariate analysis revealed that thoracic spine location (HR = 4.629, 95% CI = [1.911, 31.667], *P* = 0.042 for OS) and the lack of combined modality treatment (HR = 12.697, 95% CI = [2.664, 48.612], *P* < 0.0001 for DFS) were associated with poor survival in PSEL patients.

**Conclusions:**

PSEL demonstrates specific clinical features and is associated with a relatively good prognosis. Thoracic spine location is a significant poor prognostic factor, and combined modality treatment is associated with improved disease-free survival, but not overall survival.

**Electronic supplementary material:**

The online version of this article (doi:10.1186/s12885-017-3093-z) contains supplementary material, which is available to authorized users.

## Background

Primary spinal epidural lymphomas (PSELs) are very rare, and relatively few such tumors have been reported in the literature. Although PSEL accounts for 10% of all epidural spinal tumors [1], the epidural location is a rare site of presentation for lymphomas, characterizing only 0.1 to 3.3% of all lymphomas [2, 3]. Due to the rarity of the condition, it is difficult to enroll a sufficient number of patients with PSEL to adequately evaluate possible factors associated with survival. The largest series to date, reported by Monnard et al. [4], identified 52 patients over 20 years (1982–2002). However, the limited number of patients and the long time span have made it difficult to establish the disease parameters, such as the natural history of the disease, prognostic indicators, treatment techniques employed, and survival rates. For this study, we identified only retrospective studies, which included reports on relatively small numbers of patients or single case reports. The goal of this study was to help identify the clinical profile, treatment outcomes, and significant prognostic indicators in PSEL patients.

## Methods

### Patient characteristics

A total of 130 PSEL cases were retrospectively reviewed, including 36 Chinese patients and 94 published case reports from 1985 to 2015. Patients were selected if there was a conclusive histopathologic diagnosis of PSEL with complete clinical pathology, and there was no history of previous malignant disease or a second primary tumor. The 130 patients included 76 men (58%), 44 women (34%) and 10 unknown (8%), for a known male-to-female ratio of 1.73. The median age was 45 years (range, 3–80 years), and median follow-up was 32 months (range, 1–149 months). Clinical and pathological variables analyzed included patient age and gender; tumor stage, differentiation, location, and range; and treatment modalities employed (Table 1).

**Table 1:** Analysis of clinicopathological factors for chinese patients and report cases

Characteristics	Chinese patients	Report cases	*P* value
	*n* = 36 (%)	*n* = 94 (%)	
Median age (year)	26	48	/
Median survival (month)	12	27	/
Gender			
Male	26 (74.3)	50 (60.0)	
Female	9 (25.7)	34 (40.0)	0.640
Pathology			
B cell lymphoma	25 (78.1)	69 (74.2)	
T cell lymphoma	6 (18.8)	7 (7.5)	
Burkitt’s lymphoma	1 (3.1)	17 (18.3)	**0.035**
Stage			
I	13 (76.5)	44 (81.5)	
II-IV	4 (23.5)	10 (18.5)	0.651
Range			
1–2 spine	8 (22.9)	25 (50.0)	
3–4 spine	21 (60.0)	14 (28.0)	
≥ 5 spine	6 (17.1)	11 (22.0)	**0.010**
S + RT + CT			
Yes	5 (14.3)	45 (48.9)	
No	30 (85.7)	47 (51.1)	**<0.001**
S + CT			
Yes	17 (56.7)	57 (62.0)	
No	18 (43.3)	35 (38.0)	0.172
S			
Yes	33 (94.3)	85 (92.4)	
No	2 (5.7)	7 (7.6)	0.710
S only			
Yes	15 (42.9)	3 (3.3)	
No	20 (57.1)	89 (96.7)	**<0.001**

*S* surgery, *CT* chemotherapy, *RT* radiotherapy, Bold indicates significant values

### Treatment

Most patients diagnosed with stage IE–IIE PSEL had been treated with tumorectomy (*n* = 121, 93%); the rest had surgical contraindications or refused surgery. The primary treatment of patients consisted primarily of surgery. Individualized postoperative treatment consisted of radiation therapy alone (*n* = 26, 21%), chemotherapy alone (*n* = 20, 25%), or concurrent chemoradiation therapy (*n* = 41, 49%). The median radiotherapy dose was 40 Gy (range, 20–50 Gy), and the total dose was administered over 3–5 weeks. Twenty-eight patients (34%) had only focal treatment, whereas the remaining 55 (66%) received more than focal treatment. Eight-two patients (63%) had chemotherapy, which consisted of cyclophosphamide, doxorubicin, vincristine, and prednisone in the majority of patients; methotrexate, leucovorin, etoposide, and bleomycin were also used in some patients. Generally, 4–8 cycles of chemotherapy were administered at 3-week intervals. Methotrexate was administered in 21 patients (26%).

### Statistical analysis

The Kaplan–Meier method and log-rank tests were used to determine overall survival (OS) and disease-free survival (DFS). Independent prognostic factors for OS and DFS were identified using the Cox proportional hazards model. SPSS software (version 13.0, SPSS Inc., Chicago, IL) was used for all statistical analyses, and *P* values < 0.05 were considered statistically significant.

## Results

### Clinical features

The median patient age was 45 years (range, 3–80 years). In three of the 130 patients (2%), PSEL was detected during a routine examination. Alternatively, 112 of the patients (86%) presented with neck, back, lumbosacral, or limb pain, or with cord compression syndrome. For 15 cases (12%) reviewed from published case reports, the details of presentation were not provided. The most commonly reported symptoms were motor weakness or hypoesthesia (62%), back pain (59%), lumbosacral pain (32%), limb pain (28%), neck pain (9%), bowel dysfunction (23%), bladder dysfunction (19%), and low-grade fevers (2%). The time from the first symptom to diagnosis varied from 3 days to 5 years. Patient characteristics are presented in Additional file 1: Table S1 and Additional file 2: Table S2.

### Pathologic features

Case reviews showed tumor pathologies of B-cell lymphoma (*n* = 88, 76%), T-cell lymphoma (*n* = 13, 11%), or Burkitt’s lymphoma (*n* = 15, 13%). Subsets of cases were identified with tumor cells showing the strong expression of leukocyte common antigen (19/19, 100%) or the absence of CD20 expression (21/27, 78%). Additional immunohistochemical assays performed in a subset of the cases included detection of CD3 (8/13, 62%), CD45 (6/9, 67%), and CD79 (7/7, 100%). See Additional file 1: Table S1 and Additional file 2: Table S2.

### Survival

The median follow-up period was 32 months. Relapses were observed in 21 patients (19%) after a median period of 12 months, primarily in the central nervous system (*n* = 11), lymph nodes (*n* = 4), chest or abdomen (*n* = 5), and liver (*n* = 2). The 3-year OS and DFS were 81.1 and 46.3%, respectively.

### Prognostic factors

**Fig. 1:**
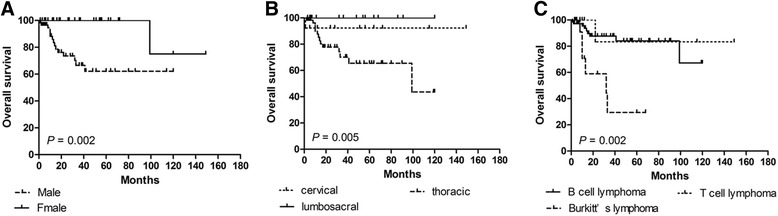
Kaplan–Meier survival curves for clinicopathologic factors of patients with primary spinal epidural lymphomas. **a** Survival curves for OS in relation to gender in primary spinal epidural lymphomas as indicated. **b** Survival curves for OS in relation to tumor location. **c** Survival curves for OS associated with different pathological factors as indicated. Favorable prognostic factors in primary spinal epidural lymphoma patients were female sex, B-cell lymphoma type, and cervical spine location

**Fig. 2:**
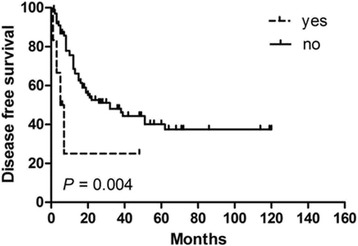
Kaplan–Meier survival curves analyze for DFS rates associated with surgery alone vs. others

**Fig. 3:**
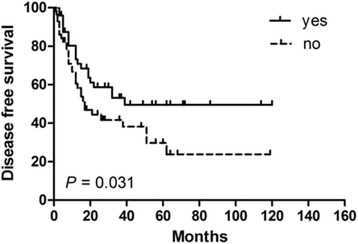
Kaplan–Meier survival curves analyze for DFS rates associated with surgery followed by chemotherapy and radiotherapy vs. others

**Fig. 4:**
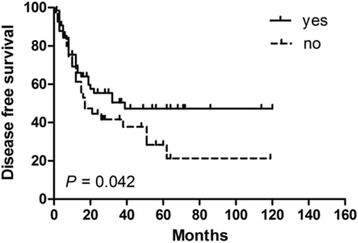
Kaplan–Meier survival curves analyze for DFS rates associated with surgery followed by chemotherapy vs. others

**Fig. 5:**
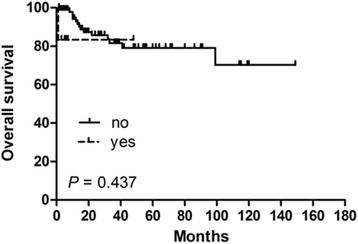
Kaplan–Meier survival curves analyze for OS rates associated with surgery alone vs. others

**Fig. 6:**
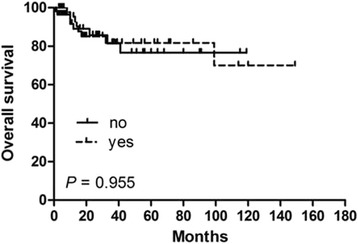
Kaplan–Meier survival curves analyze for OS rates associated with surgery followed by chemotherapy and radiotherapy vs. others

**Fig. 7:**
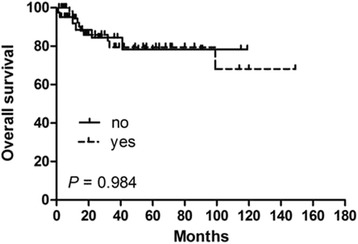
Kaplan–Meier survival curves analyze for OS rates associated with surgery followed by chemotherapy vs. others

In order to identify potential prognostic factors associated with survival in PSEL patients, various clinicopathologic variables were evaluated (Table 2). Using univariate analysis, gender, pathological pattern, tumor differentiation, tumor location, and tumor range were found to be associated with OS (*P* < 0.05), but not with DFS. The 3-year OS rates for males and females were 60.3% and 100% (*P* = 0.002), respectively (Fig. 1a). The 3-year OS rates for thoracic, cervical, and lumbosacral spine locations were 64.1, 94.3, and 100% (*P* = 0.005), respectively (Fig. 1b). The 3-year OS rates for B-cell, T-cell, and Burkitt’s lymphoma were 87.7, 83.3, and 29.5% (*P* = 0.002), respectively (Fig. 1c). In terms of treatments received, the 3-year DFS rate for patients receiving S alone was 23.3% (*P* = 0.004, compared with the other treatment groups; Fig. 2), while patients receiving S + RT + CT had a 3-year DFS rate of 49.6% (*P* = 0.031, compared with the other treatment groups, Fig. 3); patients receiving S + CT had a 3-year DFS rate of 50.4% (*P* = 0.042, compared with the other treatment groups; Fig. 4). For the same patient treatment groups, the 3-year OS rates were 80.0, 81.7, and 79.5%, respectively; these did not significantly vary (*P* > 0.05 for all; Figs. 5, 6, and 7). Multivariate analysis revealed that thoracic spine location (HR = 4.629, 95% CI = [1.911, 31.667], *P* = 0.042 for OS) and the lack of combined modality treatment (HR = 12.697, 95% CI = [2.664, 48.612], *P* < 0.0001 for DFS) were associated with poor survival in PSEL patients.

**Table 2:** The 3-year' OS and DFS rates associated with primary spinal epidural lymphomas patients

Characteristics	Case (*n*)	3 year's		3 year's	
		DFS(%)	P	OS	P
Age (year)					
< 45	64	54.8		70.5	
≥ 45	64	41.8	0.413	90.9	0.181
Gender					
Male	76	47.7		60.3	
Female	44	47.7	0.608	100.0	**0.002**
Pathology					
B cell lymphoma	88	47.6		87.7	
T cell lymphoma	13	53.6		83.3	
Burkitt’s lymphoma	15	25.0	0.081	29.5	**0.002**
Differentiate					
Poor	19	54.5		74.1	
Moderate	12	50.5		68.2	
High	8	60.0	0.309	100.0	**0.035**
Stage					
I	58	36.7		75.1	
II-IV	14	77.4	0.084	/	0.198
Location					
Cervical	14	74.0		94.3	
Thoracic	69	35.7		64.1	
Lumbosacral	25	57.4		100.0	
Sacral	3	/	0.089	/	**0.005**
Range					
1–2 spine	17	36.4		86.2	
3–4 spine	33	46.7		81.9	
≥ 5 spine	18	41.2	0.698	74.4	**0.013**
S + RT + CT					
Yes	49	49.6		81.7	
No	56	38.0	**0.031**	81.4	0.955
S + CT					
Yes	65	50.4		79.5	
No	40	41.4	**0.042**	84.3	0.984
S					
Yes	96	46.8		80.0	
No	9	47.6	0.994	100.0	0.592
S only					
Yes	5	23.3		80.0	
No	100	75.6	**0.004**	81.6	0.437

*S* surgery, *CT* chemotherapy, *RT* radiotherapy, Bold indicates significant values

## Discussion

With a study cohort of 130 patients treated over a 30-year period, the current study represents the largest one to focus on PSEL. Prior to this, the largest one reported on only 52 patients, and predated modern radiotherapy and chemotherapy protocols.

The origin of primary spinal epidural lymphomas (PSEL) remains controversial. It is known that lymphoma may arise anywhere lymphatic tissue is found. However, whether there is lymphoid tissue in the epidural space has been debated [5, 6]. Rubinstein was the first to demonstrate the presence of lymphoid cells in epidural tissue, and he introduced the theory of antigenic stimulation with a transformation cascade [7]. Some have suggested that PSEL may originate from either paraspinal, spinal, or retroperitoneal tissues, accessing the epidural space via the interspinal foramina [8, 9]. However, the occurrence of lymphoma in this location indicates that lymphoid precursor cells are present in the space. Metastasis is the most common sacral malignancy, whereas chordoma in the most common primary sacral tumor [10]. PSELs represent 10% of epidural spinal tumors, but the epidural location is a rare presenting site for lymphomas, being seen in only 0.1 to 3.3% of all lymphomas. When lymphoma has been found in the spine, it has been reported most often in the lumbar or lower dorsal area [11]. However, in this study, we found the thoracic spine to be the most common site (62%) for lymphoma.

The most common presenting symptoms reported in the literature have included lower limb weakness, localized back pain, and bladder dysfunction [8, 9]. However, in this study, the most common symptoms seen were motor weakness or hypoesthesia (62%), back pain (59%), lumbosacral pain (32%), limb pain (28%), neck pain (9%), bowel dysfunction (23%), bladder dysfunction (19%), and low-grade fevers (2%). It is noteworthy that most patients presented with persistent back pain for considerably long periods of time prior to diagnosis. Therefore, persistent back pain in a patient should be considered a warning symptom for a more serious illness.

On histopathological examination, these tumors show atypical lymphoid cell proliferation. On immunohistochemistry, tumor cells are positive for leukocyte common antigen (LCA) and CD20, but negative for CD138, CD30, and CD3. B-cell lymphoma is the most common type at this site [12], which is consistent with our findings. Our study found that tumor pathologies consisted of B-cell lymphoma (76%), T-cell lymphoma (11%), and Burkitt’s lymphoma (13%). Furthermore, tumor cells were associated with the strong expression of leukocyte common antigen (100%) and the absence of CD20 expression (78%). PSELs are almost invariably of the B-cell type, although indolent B-cell and T-cell variants are sometimes seen.

There has been no optimization of treatment strategies for PSEL patients to improve patient outcomes. Unfortunately, there is a high mortality rate within the first year of diagnosis due to early metastasis and tumor recurrences. Accordingly, the importance of aggressive, systemic therapy has been recognized over the last few decades, although there have been reports of patients being cured with local radiotherapy alone [13]. In cases of patients who present with signs of spinal cord compression, surgical intervention for tissue diagnosis and decompression is required. Aabo et al. [14] reported no difference between the outcomes in patients who underwent decompressive laminectomy and radiotherapy and those who received spinal radiation only. Chemoradiotherapy remains the mainstay of treatment because lymphomas are highly radio- and chemosensitive tumors. The CHOP regime, in particular, is still considered the gold standard for treatment. A radiotherapy dose of 3500 cGy to 4000 cGy delivered in 20–25 fractions over 3–4 weeks is required to achieve radical cure [9]. Intensive chemotherapy should also be initiated immediately following diagnosis and be delivered over a short period of time. From reviewing several studies, we found that combined modality treatment is associated with a lower recurrence rate and improved disease-free survival in PSEL, but not overall survival. Similar findings have not been reported by other researchers. In this study, the median survival time of 36 Chinese patients is 12 month. It shorter than non-Chinese cases (12 VS. 27). Optimization of treatment strategies for Chinese patients included surgery (33/35, 94.3%), surgery followed by chemotherapy (17/35, 56.7%), surgery only (15/35, 42.9%), and surgery followed by chemotherapy and radiotherapy (5/35, 14.3%). There are less Chinese patients with PSEL treated with combined modality treatment compared to non-Chinese cases because of medical expenses. Kaplan–Meier analysis revealed that combined modality treatment is associated with improved disease-free survival for all 130 PSEL cases. Therefore, to our knowledge, combined modality treatment should be considered an standardized treatment strategy for PSEL presently.

## Conclusions

The results of the present study indicate that PSEL has a relatively good prognosis, that thoracic spine location is a significant poor prognostic factor, and that combined modality treatment is associated with improved disease-free survival, but not overall survival. This study represents the largest to date on PSEL although it is limited by its retrospective design and the relatively small patient cohort. Nevertheless, our findings give important insight into this rare, challenging disease, and expand our knowledge base on this aggressive tumor.

## Additional files

Additional file 1: Table S1. The clinical and pathologic features of Primary spinal epidural lymphomas cases examined in China. (XLSX 13 kb)
Additional file 2: Table S2. The clinical and pathologic features of Primary spinal epidural lymphomas cases published. (XLSX 20 kb)

## Abbreviations

CI: Confidence interval
CT: Chemotherapy
DFS: Disease-free survival
HR: Hazard ratio
OS: Overall survival
PSELs: Spinal epidural lymphomas
RT: Radiotherapy
S: Surgery
